# Dimensions Underlying the Perceived Similarity of Acoustic Environments

**DOI:** 10.3389/fpsyg.2017.01162

**Published:** 2017-07-12

**Authors:** Francesco Aletta, Östen Axelsson, Jian Kang

**Affiliations:** ^1^Acoustics Group, School of Architecture, University of Sheffield Sheffield, United Kingdom; ^2^WAVES Research Group, Department of Information Technology, Ghent University Ghent, Belgium; ^3^Gösta Ekman Laboratory, Department of Psychology, Stockholm University Stockholm, Sweden

**Keywords:** soundscape, perceived similarity, acoustic environment, PCA, listening experiment

## Abstract

Scientific research on how people perceive or experience and/or understand the acoustic environment as a whole (i.e., soundscape) is still in development. In order to predict how people would perceive an acoustic environment, it is central to identify its underlying acoustic properties. This was the purpose of the present study. Three successive experiments were conducted. With the aid of 30 university students, the first experiment mapped the underlying dimensions of perceived similarity among 50 acoustic environments, using a visual sorting task of their spectrograms. Three dimensions were identified: (1) Distinguishable–Indistinguishable sound sources, (2) Background–Foreground sounds, and (3) Intrusive–Smooth sound sources. The second experiment was aimed to validate the results from Experiment 1 by a listening experiment. However, a majority of the 10 expert listeners involved in Experiment 2 used a qualitatively different approach than the 30 university students in Experiment 1. A third experiment was conducted in which 10 more expert listeners performed the same task as per Experiment 2, with spliced audio signals. Nevertheless, Experiment 3 provided a statistically significantly worse result than Experiment 2. These results suggest that information about the meaning of the recorded sounds could be retrieved in the spectrograms, and that the meaning of the sounds may be captured with the aid of holistic features of the acoustic environment, but such features are still unexplored and further in-depth research is needed in this field.

## Introduction

One of the first definitions of ‘soundscape’ was given in the Handbook for Acoustic Ecology (first published in 1978) – “An environment of sound (or sonic environment) with emphasis on the way it is perceived and understood by the individual, or by a society” (Truax, 1978). The concept has attracted interest from various scientific and social disciplines: acoustics, psychology, sociology, urban planning, ecology, and more. Due to its strong interdisciplinary appeal it is a field of wide experimentation. The literature in the field is growing, proposing both theoretical models and practical approaches (Schulte-Fortkamp and Dubois, 2006; Cain et al., 2009, 2013; Axelsson et al., 2010; Davies, 2013; Schulte-Fortkamp and Kang, 2013). In 2008 the International Organization for Standardization (ISO) created a new working group with the mission to develop the first International Standard on soundscape, ISO 12913. Part 1 of the standard defines ‘soundscape’ sas an “acoustic environment as perceived or experienced and/or understood by a person or people, in context” (ISO, 2014). Thus, there is a general agreement that soundscape concerns human perception of the acoustic environment. This is comparable to the European Landscape Convention that defines ‘landscape’ in similar terms (Council of Europe, 2000). Currently the ISO working group is preparing Part 2 on data collection and reporting requirements in soundscape studies, which include developing soundscape indicators (i.e., acoustic terms used to predict human responses to the acoustic environment).

In order to help European policymakers and authorities to understand and fulfill their responsibilities with regards to the protection of so called ‘quiet areas,’ the European Environment Agency (EEA) published a good practice guide in 2014 (EEA, 2014). It recommends four complementary methods for identifying quiet areas. The soundscape approach is one of them. EEA also calls for further in-depth research in this field. For example, EEA identifies a need to develop “indicators and measurements of human appreciation of quiet areas and perceived acoustic quality.” Thus, EEA provides its support to soundscape research and underlines the need of soundscape indicators.

There have been a few attempts to develop soundscape indicators by identifying relationships between soundscape and established acoustic parameters, such as A-weighted equivalent continuous sound pressure level, and psychoacoustic parameters, such as: Loudness, Roughness, Sharpness, and related percent exceedance levels (Brambilla et al., 2013; Rychtáriková and Vermeir, 2013). The latter are thought to better describes particular auditory sensations which might not be expressed by simple energetic metrics (Genuit and Fiebig, 2006). Detailed information about these three psychoacoustic parameters (including definitions and applications) are found in Fastl and Zwicker (2007). Nevertheless, this approach is not necessarily successful, because many established psychoacoustic parameters are primarily developed for the purpose of single sounds or sound sources and used within a “product sound quality” framework for industrial applications (e.g., automotive sector, domestic appliances industry, etc.). They were not developed for the purpose of soundscape, nor for measuring the acoustic environment holistically. Alternatively, some researchers (Herranz Pascual et al., 2010) have tried to incorporate the human experience of a place in a soundscape index. Yet others believe that “human responses should not be equated to acoustic measures” (Andringa et al., 2013). In fact, the soundscape methodology is far more holistic than mere noise control engineering, shifting from a quantitative to a qualitative approach to the assessment and management of the (urban) acoustic environments. Several studies have pointed out the need for more standardization with regards to these issues (Brown et al., 2011; Aletta et al., 2014). Kang et al. (2016) proposed an overview of the state-of-art in soundscape research, and the challenges this approach is facing.

**FIGURE 1:**
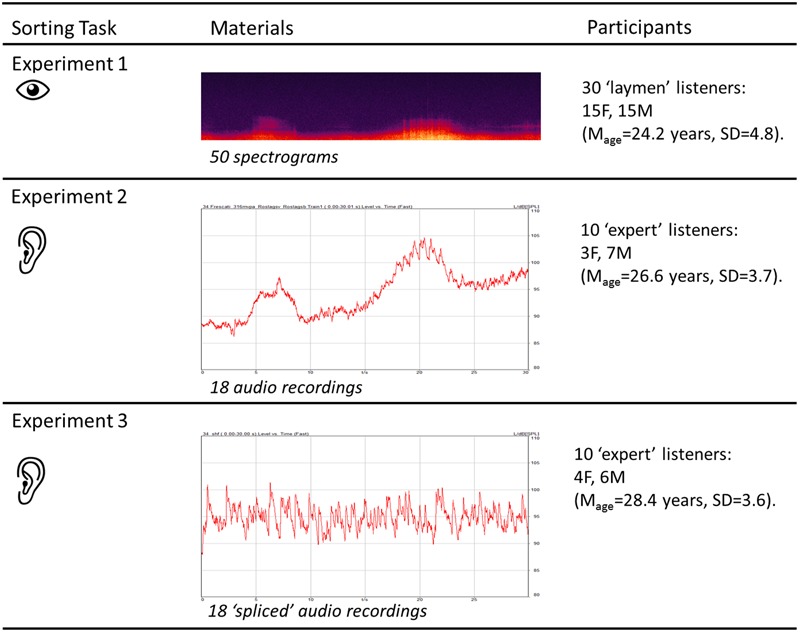
Overall experimental methodology of the study, with the different sorting tasks performed by the different groups of participants.

There is still no consensus about what acoustic properties might be meaningful for describing the perceived properties of the acoustic environments and how the former relate to the latter. Hence, the purpose of the present study was to explore the acoustic properties of acoustic environments holistically. The main research questions were: (1) whether dimensions describing perceived similarity between acoustic environments, in terms of their acoustic properties, could be identified; and (2) whether those dimensions could be satisfactorily explained by established acoustic metrics. Three successive experiments were conducted. The first experiment mapped the underlying dimensions of perceived similarity among 50 acoustic environments based on their acoustic properties. The second experiment was carried out in order to validate the results from Experiment 1 by a listening experiment. The third experiment replicated Experiment 2 with spliced signals to investigate whether the meaning of the sounds was an important factor. **Figure 1** summarizes the overall methodology of this paper, the details of which will be further discussed in the corresponding sections.

## Experiment 1: Sorting of Spectrograms

### Method

#### Participants

Thirty undergraduates and post-graduates at the University of Sheffield, 18 to 33 years old, participated in the experiment (15 women, 15 men; *M*_age_ = 24.2 years, *SD* = 4.8). The ethnic distribution of the sample was 20 ‘White or Caucasian’ and 10 ‘Asian or Pacific Islander.’ Participants were selected from a group of 100 persons who completed an online survey circulated via the established email list for student volunteers at University of Sheffield. The questions in the online survey were designed to achieve a diverse group of participants in terms of gender, age and ethnic origin. All participants had normal color vision as tested by the “Ishihara test for color deficiency” (Ishihara, 1957). Because the goal was to test only whether or not the participant had a normal color appreciation, a reduced version of the test was used. It included 6 plates, selected according to Ishihara’s instructions (Ishihara, 1957). The 30 participants who completed the experiment were rewarded for volunteering with a GBP 10 gift card.

#### Stimulus Material

**FIGURE 2:**
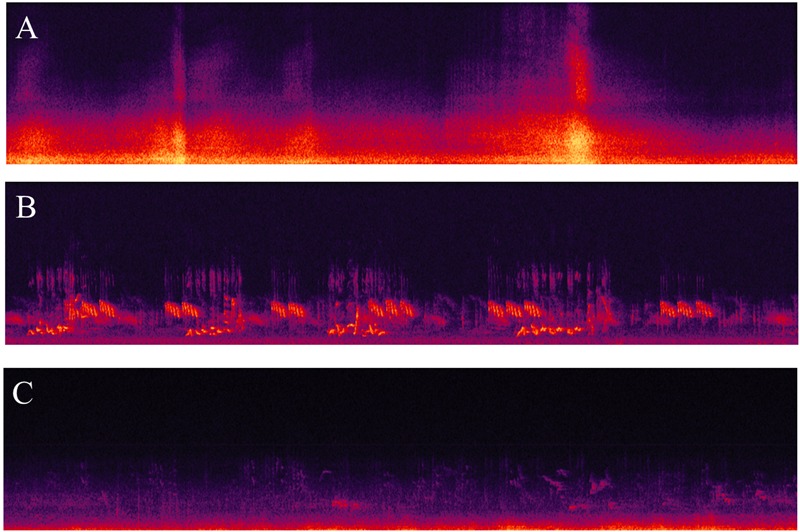
Three examples **(A–C)** of spectrograms used in Experiment 1.

Fifty recordings (30s) from Axelsson et al. (2010) were used for this experiment. They were selected from a library of binaural recordings of outdoor acoustic environments (London and Stockholm) with the aim to achieve a large variation in overall sound-pressure levels and urban/peri-urban locations. **Table 1** presents the A-weighted equivalent continuous sound pressure levels (*L*_Aeq,30s_) and the main sound sources of the 50 experimental sounds. In order to create visual representations of the acoustic data, the fifty audio files (.wav) were imported in Adobe Audition 3.0. For each binaural recording, the spectrogram (time vs. frequency) was plotted for the right channel. The spectrograms were set to have the time on the *X*-axis (0–30 s, 1 s steps) and the frequency on the *Y*-axis, with a linear scale (0–25 kHz, 1 kHz steps). Regarding the spectral controls for the color scale of the sound-pressure-level dimension, the software default settings were used (132 dB range, 512 frequency bands resolution, gamma index 2) and the three sampling colors were: yellow (RGB 254, 250, 84 – width 67%), orange (RGB 249, 47, 0 – width 76%) and purple (RGB 45, 7, 69 – width 80%). The 50 spectrograms were printed in color on glossy photo paper (18.5 × 4.5 cm, 150 dpi resolution). **Figure 2** presents three examples (Panels A–C) of the 50 spectrograms used in the experiment.

**Table 1:** Description of the 50 experimental sounds with regards to A-weighted equivalent continuous sound pressure levels (dB) and the main sound sources.

Sound	*L*_Aeq,30s_	Main foreground sound sources	Main background sound sources
1	69.03	Road traffic	Airplane, birdsong
2	54.47		Birdsong, children, train passing by
3	47.64		Voices, birdsong, road traffic
4	45.18	Birdsong	Road traffic
5	52.61	Fan	Voices
6	67.15	Motorcycle passing by	Birdsong, wind, footsteps
7	58.04	Fan	Road traffic, birdsong, dripping water
8	76.33	Road traffic, airplane	Car alarm, car horn
9	69.80	Voices, footsteps	Car horns
10	63.05	Pouring water	Road traffic, airplane
11	81.20	Road traffic	
12	50.93	Birdsong	Road traffic, hammering
13	60.13	Airplane	Birdsong, construction works
14	65.36	Road traffic	Hammering, birdsong
15	52.12	Birdsong, footsteps	Voices, children playing, road traffic
16	71.74	Voices	Road traffic
17	51.99	Footsteps, seagulls, wind, rustling leaves	Car passing by
18	77.09	Road traffic	
19	77.37	Pneumatic drill	
20	80.31	Airplane	
21	76.62	Children playing	
22	68.31	Waterfall	Birdsong
23	69.99	Airplane	Birdsong
24	74.08	Children playing	Road traffic, angle grinder
25	60.18	Train passing by	Road traffic, birdsong, footsteps
26	72.74	Fountain	Voices, road traffic
27	51.62	Wind, rustling leaves	
28	72.68	Airplane, road traffic, street sweeper	
29	71.03	Children playing	Road traffic
30	74.44	Angle grinder, road traffic	
31	63.14	Children playing	Road traffic, birdsong, bell
32	73.69	Road traffic	Car alarm, car horn
33	67.09	Road traffic, airplane	Birdsong
34	78.91	Road traffic, train passing by	Birdsong
35	60.93	Rain	Road traffic, voices
36	44.16		Fan
37	72.71	Fountain, ambulance	Reversing lorry
38	72.88	Children playing	
39	57.74	Birdsong, voices	Road traffic
40	45.73	Birdsong	Ambulance, Airplane
41	76.23	Train passing by	Road traffic, birdsong
42	67.97	Footsteps, road traffic	
43	67.40	Birdsong	Road traffic
44	74.27	Road traffic, Boing 747 landing	
45	63.15	Fountain	Road traffic, voices
46	61.91	Birdsong	Road traffic, recordist hushing
47	56.53	Footsteps	Road traffic, wind, birdsong
48	54.00	Dog playing in water	Road traffic
49	63.65	Fountain, airplane	Voices, birdsong
50	70.29	Chainsaw	Voices, road traffic

#### Design and Procedure

The experiment took place in an office room at the School of Architecture, University of Sheffield. The design of the experiment consisted of a two-stage data collection procedure: sorting and interview. The participants took part individually. First, the color vision test was performed for each participant. Successful participants were admitted to the following stage. One participant was omitted due to partial color-blindness.

Seated at an office desk, every participant was provided with the 50 color prints of the spectrograms as a stack of photographs mixed in a unique irregular order for each participant. Importantly, they were not informed about what the photographs depicted or what spectrograms represent (i.e., acoustic properties of the recorded acoustic environments). Thus, the participants were expected to treat the photographs as any abstract images, and were instructed to sort the prints into mutually exclusive groups according to the similarity of the images, and in as many groups as they wanted (2 being the minimum and 25 the maximum). In addition, they were asked to pay attention to whether or not they developed any specific sorting criteria. This information was required in the subsequent interview. Participants were allowed to revise their sorting throughout the experimental session, including the interview.

After completing the sorting task, the participants were interviewed, with the purpose to learn whether or not they had developed any soring criteria, and then which they were. This information was used to interpret the sorting results. During the interview the experimenter took notes (cf. Axelsson, 2007). The 30 experimental sessions lasted between 8 and 45 min each (*M*_time_ = 19.5 min, *SD* = 8.9). There were no time restrictions.

### Results

The participants created between 3 and 17 groups of spectrograms (*M* = 8.0 groups, *SD* = 3.7). The sorting data was used to create a proximity matrix based on how often all possible pairs of the 50 spectrograms appeared in the same group, summed over all 30 participants (cf. Axelsson, 2007). The proximity matrix was subjected to MDS (SPSS 21 for Windows). By using the ALSCAL technique (Young and Lewyckyj, 1979), six solutions, with one to six dimensions (stress values: 0.488, 0.257, 0.156, 0.109, 0.088, 0.071), were extracted (Coxon, 1982). Based on a ‘scree’ criterion (Cattell, 1966) the three-dimensional solution was selected for further analysis.

**FIGURE 3:**
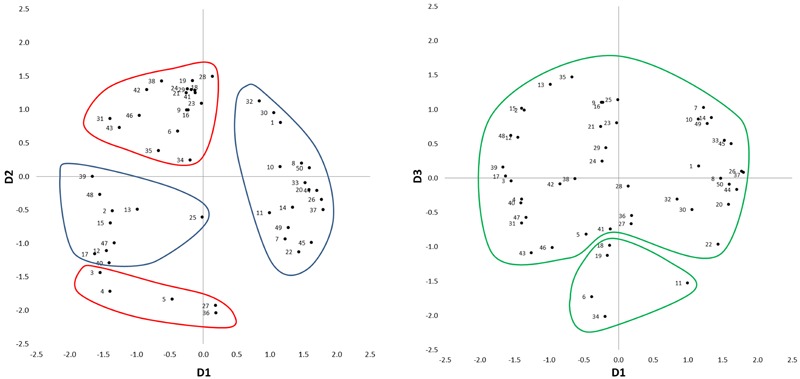
Three-dimensional MDS solution for Experiment 1. On the left plot: the blue clusters D1 gather *distinguishable* vs. *indistinguishable* sound sources, while the red clusters on D2 gather *background* vs. *foreground* sounds. On the right plot: the green clusters on D3 gather *intrusive* vs. *smooth* sound sources.

**Figure 3** presents the three-dimensional MDS solution. Data points represent the 50 spectrograms, numbered in agreement with **Table 1**. In order to aid the interpretation of the three dimensions, the first author created clusters of spectrograms through visual inspection of the spectrograms and by listening to the corresponding audio recordings. In the listening sessions he sought a holistic listening style, aiming to disregard the semantic content, because it was assumed that the information about the ‘meaning’ of the sources was not available to the participants in sorting the spectrograms.

The first cluster contained spectrograms with positive values in the first dimension (D1). In the interviews they were often described as *“dominated by horizontal stripes,” “representing all range of colors”* or *“with colors blurring into each other*.*”* Auditory inspection revealed sounds similar to white noise. Typical dominant sound sources were fountains (e.g., Sounds 26 and 37), road traffic (e.g., Sounds 8 and 33), and aircraft (e.g., Sound 20). Combinations of several noisy sources, often affecting wide frequency ranges, typically provided an acoustic environment where different auditory features were *indistinguishable*.

The second cluster contained spectrograms with negative values in D1. In the interviews they were often described as having *“spikes,” “mostly vertical shapes,”* and *“noticeable patterns*.*”* Auditory inspection revealed clearly identifiable sound sources against a generally quiet background: footsteps (e.g., Sounds 17 and 47), birdsong (e.g., Sounds 12 and 15), and a dog playing in the water (Sound 48). Thus, the second cluster represented acoustic environments where the sound sources were *distinguishable*. Consequently, D1 was interpreted as to represent Distinguishable–Indistinguishable sound sources.

The third cluster had positive values in the second dimension (D2) and contained spectrograms that were referred to as *“yellow”* or *“deep red*.*”* Contrariwise, the fourth cluster contained spectrograms with negative values in D2, referred to as *“purplish”* or *“dark*.*”* This suggested that D2 was related to sound-pressure level. Auditory inspection of the corresponding audio files revealed that D2 was associated with distance of the sound sources from the listener. The third cluster represented *foreground* sounds, where sound sources were close (e.g., Sounds 38 and 42); whilst the fourth cluster represented *background* sounds, where sound sources were distant (e.g., Sounds 5 and 27). As a result, D2 was interpreted as to represent Background–Foreground sounds.

For the third dimension (D3), two separate clusters were created. The first of these two clusters contained spectrograms with negative values in D3. These spectrograms were described as *“eventful”* with *“things going on”* and *“aggressive*.*”* The second of the two clusters contained spectrograms with mainly positive values in D3. They were considered as *“even,” “smooth,”* and *“generally flat*.*”* In the first case, sounds were characterized by an *intrusive* source, temporarily dominating the acoustic environment (e.g., Sounds 6 and 19). In the second case, sounds were *smooth* and organic, regardless of the temporal or spectral features (e.g., Sounds 7 and 35). The perception was that, regardless of the semantic content of the excerpts and their spectral content, no sound sources were being added to the sound field and this was evolving in time in an even way; D3 was therefore interpreted as to represent Intrusive–Smooth sound sources.

With the intention to provide further material for the interpretation of the three dimensions, the acoustic signals that correspond to the 50 spectrograms were subjected to acoustic analyses. For each acoustic signal (30s) a set of 100 acoustic and psychoacoustic parameters were calculated. This included unweighted, A-weighted and C-weighted equivalent continuous sound pressure levels (*L*_eq_, *L*_Aeq_, *L*_Ceq_), Loudness (*N*), Sharpness (*S*), Roughness (*R*), Fluctuation strength (*Fls*), Tonality (*Ton*), percent exceedance levels for the above mentioned parameters (*P*_1_, *P*_5_, *P*_10_, *P*_25_, *P*_50_, *P*_75_, *P*_90_, *P*_95_, *P*_99_), a measurement of the spectral variability (*L*_Ceq_–*L*_Aeq_), and the measurements of the temporal variability (*P*_1_–*P*_99_, *P*_5_–*P*_95_, *P*_10_–*P*_90_, *P*_25_–*P*_75_). The rationale for doing this is that there are several studies (Botteldooren et al., 2006; De Coensel and Botteldooren, 2006) in soundscape research suggesting that the way humans construct their auditory perceptual dimensions can be related to three main ‘physical features’ of the auditory stimuli: the intensity, the spectral content and the temporal structure of sounds. Hence, it seemed reasonable to test a large set of psychoacoustic metrics (which are expected to account for intensity and spectral content) and an equally large combination of differences of their percent exceedance levels (which are expected to account for different degrees of temporal variability).

Data screening revealed curvilinear relationships between the three dimensions and some of the acoustic and psychoacoustic parameters. For this reason the base-10 logarithms were calculated for all of the 100 parameters, except for six of them that included negative values.

Three stepwise multiple linear regression analyses were conducted, using D1, D2, and D3 as dependant variables and the complete set of 194 parameters as independent variables (SPSS 21 for Windows). The strongest predictors for the models of D1 (*F*_4,45_ = 42.79, *p* < 0.001, *R*^2^ = 0.79), D2 (*F*_5,44_ = 37.07, *p* < 0.001, *R*^2^ = 0.81) and D3 (*F*_3,46_ = 9.81, *p* < 0.001, *R*^2^ = 0.39) are reported in **Table 2**.

**Table 2:** The three stepwise linear regression models computed for D1, D2, and D3, with the best predictors, and the corresponding unstandardized coefficients (β), *t* and *p*-values.

Model	Predictors	β	*t*	Sig.
D1	*L*_A50_	0.702	9.89	*p* < 0.001
	Log(*S*_1_–*S*_99_)	–0.937	–3.85	*p* < 0.001
	Log(*Fls*_25_–*Fls*_75_)	–0.236	–2.45	*p* = 0.018
	Log(*S*_10_–*S*_90_)	0.531	2.28	*p* = 0.028
D2	Log(*N*_1_–*N*_99_)	0.544	6.07	*p* < 0.001
	Log(*Fls*_95_)	0.384	4.03	*p* < 0.001
	Log(*S*_1_)	0.361	3.67	*p* = 0.001
	*Fls*_10_	–0.328	–3.33	*p* = 0.002
	*Fls*_99_	–0.174	–2.33	*p* = 0.024
D3	*L*_A10_–*L*_A90_	–1.241	–4.57	*p* < 0.001
	Log(*L*_A25_–*L*_A75_)	0.810	3.00	*p* = 0.004
	*Fls*_99_	–0.246	–2.10	*p* = 0.041

*L*_A50_ explained 38.9% of the variance in D1. When controlling for this variable, log measurements of variability in Sharpness [Log(*S*_1_–*S*_99_)] explained an additional 34.9% of the variance. The positive relationship between D1 and *L*_A50_ shows that there was more acoustic energy associated with the sounds interpreted as indistinguishable, compared to the sounds interpreted as distinguishable. This indicates that, in the former case, several sound sources were present, possibly masking each other. It seems reasonable that several sound sources are louder than one. The negative relationship between D1 and Log(*S*_1_–*S*_99_) shows that as the variability in Sharpness increased, sounds were interpreted as all more distinguishable.

D2 was strongly and positively associated with variability in loudness levels Log(*N*_1_–*N*_99_), which alone explained 66.6% of the variance in D2. This positive relationship indicates that there is a larger variability in Loudness in sounds interpreted as to represent the foreground than in sounds interpreted as to represent the background. This seems plausible, because background sounds at a distance would not vary much in loudness.

D3 was chiefly associated with variability in A-weighted sound-pressure levels: *L*_A10_–*L*_A90_ and Log(*L*_A25_–*L*_A75_), which explained 21.5 and 11.7% of the variance in D3, respectively. However, the two parameters work in opposite directions, where the former had a negative relationship and the latter a positive relationship with D3. This information is not particularly helpful in moving forward with the interpretation of D3. Thus, the regression analyses resulted in meaningful information for dimensions D1 and D2.

### Discussion

The purpose of Experiment 1 was to map the underlying dimensions of the acoustic properties of acoustic environments considered holistically. Measures of perceived similarity of 50 spectrograms were subjected to MDS analysis. Three dimensions were identified: (D1) Distinguishable–Indistinguishable sounds sources, (D2) Foreground–Background sounds, and (D3) Intrusive–Smooth sound sources. Stepwise multiple linear regression analyses with D1, D2 and D3 as dependent variables and 194 acoustic and psychoacoustic parameters as predictors showed that D1 was positively associated with *L_A50_* and negatively associated with Log(*S_1_–S_99_*). D2 was positively associated with Log(*N_1_–N_99_*). D3 was mainly associated with variability in A-weighted sound-pressure levels, but the percentage of explained variance was low. For this reason it was not worthwhile to give D3 any further attention.

The importance of fore- and background sounds, as well as distinguishable and indistinguishable sounds has been raised previously (Andringa, 2013; Andringa and van den Bosch, 2013). Andringa (2013) argues that these are central dimensions of soundscape and perceived safety. A close or indistinguishable sound source may induce a feeling of threat, whereas a distant or distinguishable sound source may induce a feeling of control.

It is interesting that none of the dimensions (D1–D3) were well-predicted by any single acoustic or psychoacoustic parameter. In all cases a combination of at least two parameters was needed to reach a sizable percentage of variance explained in the dependent variable. This result provide support for the statement in the introduction that acoustic and psychoacoustic parameters are developed for the purpose of single sounds or sound sources, not for the purpose of soundscape, nor for measuring acoustic environments holistically.

The rationale for the method used in Experiment 1 is that spectrograms represent all acoustic information of an acoustic environment, except the phase angle of the frequencies. Thus, spectrograms were used as a tool for visualizing the acoustic data representing the 50 investigated acoustic environments. By visual inspection of the spectrograms, it was possible to decide to what degree they resembled each other. Spectrograms that look similar should represent acoustic environments that are similar. Consequently, the dimensions that underlie the similarity perceived among the spectrograms should represent holistic acoustic properties. These dimensions can be identified by the aid of Multidimensional Scaling (MDS). Furthermore, the visual sorting task allowed the participants to see and to assess the whole set of stimuli, and to fully compare them with each other.

It is reasonable to ask how many stimuli are necessary to properly map all relevant acoustic dimensions of acoustic environments. The theory behind MDS states that at least nine stimuli are needed to reach a definite MDS solution (Coxon, 1982). SPSS can handle 100 stimuli at most. The stimuli must also be selected to vary with regards to all relevant aspects. For this reason a wide selection is desirable. As specified in the method section, the 50 stimuli used in the present study represent a wide selection of acoustic environments in and around two large cities, which meet the requirements (Axelsson et al., 2010).

With regards to the quality of the present study, it could be argued that it would have been better to calculate the similarity of the spectrograms mathematically, rather than conducting an experiment based on visual perception. However, mathematical calculation of the similarities would have to be based on criteria defined by the experimenter, which could introduce a bias. Using the average response of human participants who unguided develop their own criteria in a sorting task based on what they can see in the spectrograms, and on what makes sense to them, overcomes this potential limitation.

## Experiment 2: Sorting of Audio Recordings

Considering the outcomes of Experiment 1, it is reasonable to ask to what extent Dimensions 1–3 correspond to how people perceive the acoustic environments. For this reason, a second experiment was conducted in which a new group of participants sorted a subset of the audio recordings.

### Method

#### Participants

Ten expert listeners, 22–32 years old (3 women, 7 men; *M*_age_ = 26.6 years, *SD* = 3.7), post-graduates at the Department of Music or the Acoustics Group at the School of Architecture, University of Sheffield, took part in the experiment. Two out of ten persons had also taken part in Experiment 1. Participants attended on a voluntary basis and were not reimbursed.

#### Stimulus Material

Based on the MDS solution obtained in Experiment 1, the audio files corresponding to the six most extreme spectrograms (three from the positive, and three from the negative pole) of each of the tree MDS dimensions (D1, D2, and D3 in **Figure 2**) were selected. Thus, there were 18 experimental sounds in total: Sound 17, 39, 48 (D1^-^); 26, 37, 44 (D1^+^); 5, 27, 36 (D2^-^); 19, 28, 38 (D2^+^); 6, 11, 34 (D3^-^); 13, 25, 35 (D3^+^) (see also **Table 1**).

#### Equipment

The equipment consisted of a laptop (Asus, Realtek Audio soundcard), and a pair of acoustically open, circumaural headphones (Sennheiser HD 558). The selected audio recordings were played back at the authentic sound-pressure level (Brüel & Kjær Type 4231 sound calibrator).

#### Procedure and Design

The experiment took place in the anechoic chamber of the School of Architecture, University of Sheffield. The design consisted of a two-stage data collection procedure: sorting and interview. The participants took part individually.

The experiment was designed to test whether or not the participants would reproduce the six groups that the 18 experimental sounds were selected from. Consequently, the participants were instructed to sort the 18 experimental sounds, presented in the form of icons on a computer screen, into six groups, with the restriction that there had to be exactly three sounds in each group. The sorting had to be based on the similarity of the sounds, so that similar sounds were grouped together. The participants were instructed to engage in holistic listening and assess the similarity of the sounds based on an overall sonic impression, disregarding semantic information. The experimental sounds were presented in a unique random order to every participant. The participants were allowed to play each sound as many times as desired and to revise their sorting throughout the experimental session, including the subsequent interview. Thus, after completing the sorting task, the participants were interviewed about their own sorting criteria. The 10 listening sessions lasted between 20 and 37 min each (*M*_time_ = 30.2 min, *SD* = 4.8). There were no time restrictions.

### Results

**Table 3** presents the number of complete, partially complete and incomplete groups that the 10 participants achieved. Two of the participants reproduced the six groups completely. Both were female music students. Two participants reproduced four of the six groups and the remaining two groups partly by ‘misallocating’ one sound in each. Both were post-graduates in acoustics. One participant reproduced one group completely and three groups partly. The remaining five participants reproduced 1–5 groups partly and none completely.

**Table 3:** Experiment 2: number of complete, partially complete and incomplete groups that 10 participants achieved.

Participant	Complete	Partial	Incomplete
1		1	5
2		2	4
3		3	3
4		4	2
5	1	3	2
6		5	1
7	4	2	
8	4	2	
9	6		
10	6		
Total	21	22	17

*Complete means that all three experimental sounds that belong in the same group were grouped together. Partial means that 2 out of 3 experimental sounds that belong in the same group were grouped together as expect.*

Eighteen sounds can be organized in 18! (i.e., eighteen factorial) permutations. There is 3!^6^ × 6! ways of achieving six complete groups. The probability of achieving six complete groups in the sorting task is 3!^6^ × 6!/18!, which equals 5.25 × 10^-9^. Thus, it is highly improbably to achieve six complete groups out of pure chance. Still, two participants achieved this result, independently.

To further investigate how likely it is to obtain the results reported above by pure chance, a Monte Carlo experiment was set up. In this experiment, 6 groups of 3 items were sorted at random 10 times, representing 10 participants. For each ‘participant,’ the six groups were classified as Complete, Partial or Incomplete, counted and recorded. The procedure was repeated 1,000 times. The results show that, on average, 10 participants would together achieve 0.48 complete, 19.75 partially complete, and 39.77 incomplete groups, by chance. For Experiment 2, the result was 21 complete, 22 partially complete, and 17 incomplete groups (**Table 3**). A Chi-Square test shows that the empirical results deviate statistically significantly from chance (χ^2^_2_ = 886.7, *p* < 0.001).

### Discussion

It seems that when the 10 expert listeners sorted the 18 experimental sounds in Experiment 2, six of them did something qualitatively different from the 30 participants in Experiment 1 when they sorted the 50 spectrograms. This seems to indicate that perception of acoustic environments chiefly belongs to a different domain compared to the acoustic properties of the same acoustic environments. Thus, dealing with acoustic environments it is necessary to decide if it is the perceived properties that are of interest or the acoustic properties. The two must not be confused. These results are in line with previous findings in soundscape research.

Guastavino (2007) investigated the way in which people categorize environmental sounds in their everyday lives, through a free categorisation task with open-ended verbal descriptions. The presence of human activity emerged as a main clustering criterion, suggesting that environmental sounds are processed and categorized based on their meaning, when such information is available. This seems to be the case in the present Experiment 2, but not in Experiment 1. This is also a potential limitation in the design of Experiment 2. Aucouturier and Defreville (2009) used manipulated (‘spliced’) acoustic signals, where sound sources were not identifiable, and found that individuals were still able to judge the similarity of such acoustic signals in a meaningful way. This is probably similar to what the 30 participants did in Experiment 1.

## Experiment 3: Sorting of Spliced Signals

In order to investigate whether the meaning of the sounds affected the results of the sorting task in Experiment 2, a third experiment was conducted. In this listening experiment spliced signals were used in agreement with Aucouturier and Defreville (2009).

### Method

#### Participants

Ten expert listeners, 24–33 years old (4 women, 6 men; *M*_age_ = 28.4 years, *SD* = 3.6), post-graduates at the Department of Music or the Acoustics Group at the School of Architecture, University of Sheffield, took part in the experiment. None had taken part in Experiments 1 or 2. Participants attended on a voluntary basis and were not reimbursed.

#### Stimulus Material

The same 18 sounds as used in Experiment 2 were used in Experiment 3. However, for Experiment 3 the acoustic signals were spliced in agreement with Aucouturier and Defreville (Coxon, 1982). Every signal was cut into segments of 50 ms, which then were reorganized in a unique random order.

#### Equipment, Procedure, and Design

The same equipment as in Experiment 2 was used. The procedure and design was the same as in Experiment 2. The 10 listening sessions lasted between 24 and 38 min each (*M*_time_ = 31.6 min, *SD* = 4.6). There were no time restrictions.

### Results

**Table 4** presents the number of complete, partially complete and incomplete groups that the 10 participants achieved. Two participants achieved five partial and one complete groups. One participant achieved one complete, one partial, and four incomplete groups. The remaining seven participants achieved 3–5 partial groups. A Chi-Square test comparing these results with results expected by chance (see Experiment 2 above), showed that the results deviates statistically significantly from chance (χ^2^_2_ = 40.84, *p* < 0.001).

**Table 4:** Experiment 3: number of complete, partially complete and incomplete groups that 10 participants achieved.

Participant	Complete	Partial	Incomplete
1	1	1	4
2		3	3
3		3	3
4		4	2
5		4	2
6		4	2
7		4	2
8		5	1
9	1	5	
10	1	5	
Total	3	38	19

*Complete means that all three experimental sounds that belong in the same group were grouped together. Partial means that 2 out of 3 experimental sounds that belong in the same group were grouped together as expect.*

Comparing these results with those obtained in Experiment 2 also shows a statistically significant difference between the two results (χ^2^_2_ = 17.88, *p* < 0.01). Taken together, the results indicate that the 10 participants in Experiment 2 performed better than the participants in Experiment 3. The participants in Experiment 2 achieved 21 complete, 22 partially complete and 17 incomplete groups, compared with the 3 complete, 38 partially complete, and 19 incomplete groups that the participants in Experiment 3 achieved (**Tables 3, 4**). Thus, the participants in Experiment 3 achieved fewer complete and more partially complete groups than the participants in Experiment 2. In addition, the Chi-Square coefficients show that the participants in Experiment 2 deviated more strongly from chance performance than the participants in Experiment 3.

### Discussion

In Experiment 3, a groups of expert listeners, equivalent to the participants in Experiment 2, achieved a statistically significantly worse result when listening to spliced signals, compared to the results that the participants in Experiment 2 achieved by listening to the authentic acoustic signals. Contrary to expectation and initial assumptions, these results indicate that the spectrograms include information about the meaning of the recorded sounds, not merely meaningless acoustic data.

## General Discussion and Conclusion

The purpose of the present study was to explore the acoustic properties of acoustic environments holistically. In Experiment 1, spectrograms corresponding to different urban acoustic environments were sorted based on how similar they were. The sorting data was subjected to MDS analysis, and three MDS dimensions were identified: (D1) Distinguishable–Indistinguishable sounds sources, (D2) Foreground–Background sounds, and (D3) Intrusive–Smooth sound sources. None of these dimensions were well-predicted by any single acoustic or psychoacoustic parameter. According to the experimenters’ original research plan, Experiment 2 was meant to validate the results of Experiment 1. However, only four of the ten participants achieved the expected result. This raised the question whether or not the spectrograms include information about the meaning of the recorded sounds. Consequently, a new listening experiment was conducted in which ten participants listened to and sorted spliced acoustic signals. Experiment 3 provided a statistically significantly worse result than Experiment 2. These results suggest that there is information about the meaning of the recorded sounds in the spectrograms, and that the meaning of the sounds may be captured with the aid of holistic features of the acoustic environment. These new, unknown, features remain to be discovered. A possible feature could be the ‘noticeability’ of events and/or sources. In soundscape research this has often been referred to as ‘saliency’ of the sounds (Oldoni et al., 2013), which can be defined as the likeliness of a sound event to attract the auditory attention of a listener at unconscious (i.e., biological) level. This can also be applied to the visual domain and would justify how participants were able to attribute ‘meaning’ to patterns in the spectrograms (e.g., the pneumatic drill in excerpt 19 or the birdsong in excerpt 43). To a large extent, saliency of sources would be lost in spliced signals, which is consistent with the worse performance in Experiment 3 compared to Experiment 2.

Regarding the acoustic properties of the acoustic environments, the main conclusions from this study are related to the results of Experiment 1:

(1) Sound sources interpreted as distinguishable had a lower median sound level (*L*_A50_) and a higher variability in Sharpness [Log(*S*_1_–*S*_99_)] than sound sources interpreted as indistinguishable. Thus, “distinguishable” sound sources seem to be related to acoustic environments with relatively low mean sound levels and large changes in the spectral content between low and high frequencies over time.
(2) Sounds interpreted as background had a low variability and sounds interpreted as a high variability in Loudness [Log(*N*_1_–*N*_99_)]. Thus, acoustic environments with larger loudness variability over time seem to be related to sounds that emerge from the background noise, perceptually.

Taken together, the results of this study show that at present there are no acoustic indicators available that can be used to assess acoustic environments holistically. More specifically, in the linear regression models, none of the considered acoustic metrics alone explained a large amount of variance in the dimensions underlying the perceived similarity of acoustic properties of the investigated acoustic environments. This gap has also been acknowledged by previous research, where it was pointed out that more predictive models for perceptual features are desirable in soundscape research (Aletta et al., 2016). Further in-depth research is needed in this field, which has to include mathematical modeling of the acoustic properties of acoustic environments considered holistically.

A potential limitation in this study is related to the Fast Fourier Transform (FFT) that underlie the spectrograms. The question is how different a spectrogram would be if different settings for the time, frequency and/or amplitude resolution were used, and how this would affect the results of the study. Would spectrograms that were similar in this study—using the default settings—be more or less similar if a different resolution was used? Further studies are needed to validate the present approach to the acoustic properties of the acoustic environment considered holistically.

## Ethics Statement

This study was carried out in accordance with the recommendations of Ethics Policy Governing Research Involving Human Participants, Personal Data and Human Tissue of the University of Sheffield with written informed consent from all participants. All participants gave written informed consent in accordance with the Declaration of Helsinki. The protocol was approved by the Ethical Committee of the School of Architecture of the University of Sheffield.

## Author Contributions

All authors conceived the study and designed the experiments. FA carried out the experiments. FA and ÖA analyzed the results. All authors wrote and critically reviewed the paper.

## Special Note

Portions of this work were presented in “Toward acoustic indicators for soundscape design,” Proceedings of Forum Acusticum, Krakow, Poland, 7–12 September 2014.

## Conflict of Interest Statement

The authors declare that the research was conducted in the absence of any commercial or financial relationships that could be construed as a potential conflict of interest.
